# Healthcare Professionals' Perceptions of the Impact of Active and Receptive Music Therapy and Its Continued Use in Hospital Settings

**DOI:** 10.1002/nop2.70934

**Published:** 2026-10-22

**Authors:** Román‐Carlos Rodríguez Rodríguez, Ana Noreña Peña, Teresa Chafer Bixquert, Javier González de Dios, Carmen Solano Ruiz

**Affiliations:** ^1^ Nursing Department, Health Sciences Faculty University of Alicante Alicante Spain; ^2^ Music and Music Therapy Department N3 Music Centre Alicante Spain; ^3^ Sculpture Department, Faculty of Fine Arts Polytechnic University of Valencia Valencia Spain; ^4^ Department of Paediatrics General University Hospital of Alicante, Miguel Hernández University Alicante Spain

**Keywords:** healthcare professionals, implementation, music therapy, perceptions, qualitative research

## Abstract

**Aim:**

To explore healthcare professionals' perceptions after experiential music therapy sessions, focusing on perceived personal benefits, potential clinical applications, and implementation in hospital settings.

**Design:**

A qualitative descriptive–exploratory study.

**Methods:**

Two focus groups were conducted with 22 healthcare professionals who participated in active and receptive music therapy sessions in November 2023. Data were analysed using qualitative content analysis.

**Data Sources:**

Data were generated from two focus groups conducted at the University of Alicante, Spain.

**Results:**

Two themes were identified: perceived benefits and clinical application of music therapy; and implementation and use in hospital settings. Participants described increased emotional awareness, self‐reflection, and interpersonal connection and highlighted institutional support and qualified music therapists as important for implementation.

**Conclusion:**

Healthcare professionals perceived personal benefits and viewed music therapy as a complementary intervention with potential to promote emotional wellbeing and more humanised care.

**Implications for the Profession and/or Patient Care:**

Experiential music therapy may support healthcare professionals' wellbeing and inform its integration into hospital care.

**Impact:**

Direct experience shaped professionals' understanding of music therapy and its potential clinical integration. Findings may inform nursing practice, hospital managers, and implementation research.

**Reporting Method:**

The study was reported in accordance with the Consolidated Criteria for Reporting Qualitative Research (COREQ).

**No Patient or Public Contribution:**

No patients or members of the public were involved; study participants were healthcare professionals.

## 1 Introduction

On the one hand, music therapy (MT) in hospital settings positively impacts the health and wellbeing of everyone involved, including patients, families, and healthcare staff (Facchini and Ruini 2021; Rodríguez‐Rodríguez et al. 2022; Stegemann et al. 2019; Tucquet and Leung 2014). On the other hand, hospitalisation and illness have negative cognitive and psychosocial repercussions on the quality of life of patients. These consequences are also reflected in the relationships patients maintain, for example, with their families or with healthcare personnel (Li et al. 2019). In short, hospitalisation negatively affects the way people cope with their illness because it exacerbates emotions and feelings, which can increase symptoms related to depression, anxiety, and stress (Alzahrani 2021). In this sense, the implementation of complementary therapies based on artistic interventions has been shown to be beneficial by improving healthcare while also representing a useful method at the therapeutic level that allows patients to share their feelings and experiences about their illness (Carter et al. 2023; Kennedy et al. 2014). Furthermore, this type of therapy improves the wellbeing and quality of life of patients because it encourages them psychologically, reducing the anxiety or stress caused by the course of their illness and improving social relationships and, for example, their self‐perceptions (Hu et al. 2021).

If we consider MT as a complementary therapy used to improve the hospital environment and wellbeing of patients and professionals alike, we must first consider its definition by the World Federation of Music Therapy (WFMT). MT is a professional discipline that uses music‐based experiences, including listening, singing, playing instruments, and improvisation, to promote health, wellbeing, capacity, and development at personal and social levels; it is delivered by trained professionals within a therapeutic relationship (World Federation of Music Therapy 2026). In hospital settings, it contributes to more humanised care, offering patients a non‐pharmacological method for emotional expression and regulation, as well as for connecting with their environment, thereby complementing conventional medical interventions (Stegemann et al. 2019).

It should be noted that the field of MT has been developing since the middle of the 20th century in countries including the United States of America, Canada, Argentina, Austria, the United Kingdom, and Australia. Within these countries, the law recognises this activity as a legally regulated profession, indicating the requirements a person must meet to practice MT by, for example, having a master's degree or having completed a given number of hours of professional development (European Music Therapy Confederation, n.d.). However, in other countries, including Spain, MT still lacks such regulation (Kern and Tague 2017; March‐Luján 2022).

Research has shown that healthcare professionals find MT interventions useful because, when implemented with patients at the hospital level, they improve self‐esteem (Bradt et al. 2021), cognitive function (Moreno‐Morales et al. 2020), functional communication (Liu et al. 2022), and promote and benefit both motor coordination (Cibrian et al. 2020) and communication between healthcare staff and patients, thereby helping to reduce stress and anxiety (Umbrello et al. 2019; Wilson et al. 2015). Other benefits noted in previous research are that MT encourages distraction and provides relaxation and emotional support, making it an important ingredient that allows patients to cope with their treatments while hospitalised (Silverman and Bibb 2018; Uggla et al. 2019). Work has also shown that the perceptions of MT by healthcare professionals are generally positive, with most considering that it improves the care of the psychosocial needs of patients (Lane et al. 2019). MT has also been shown to positively impact the work environment and improve the wellbeing of healthcare professionals (Ford et al. 2018).

In this sense, Silverman and Bibb (2018) recorded the perceptions of healthcare professionals regarding the value of MT in hospital environments, reporting evidence in support of its use as a therapy complementary to other existing interventions. However, their findings also highlighted that healthcare professionals had insufficient knowledge about MT and suggested that more training and incorporation of MT into hospital programmes was required. The latter finding was also indicated in two other studies carried out with nurses, which concluded that training in MT was important for its subsequent use in hospital environments, with the participants also showing interest in learning about MT and expressing positive attitudes regarding this type of therapy (Meghani et al. 2020). Furthermore, in addition to being a clinically viable therapy, the use of MT in healthcare fields is also an economically feasible option because, for example, it can lead to the use of fewer medications (Livingston et al. 2014; Trainor 2019).

Current evidence suggests that healthcare professionals recognise the potential benefits of music therapy and hold positive attitudes towards its use in healthcare settings (Chadder 2019; Biard et al. 2026; Giordano et al. 2025). Recent research has shown that nurses consider music‐based interventions to be a valuable resource for patient care, although they identify practical and organisational barriers that may hinder their implementation (Borick et al. 2026). Building on this evidence, the present study explores healthcare professionals' perceptions following their participation in a structured active and receptive music therapy session. This experiential approach responds to the need for further evidence on how direct exposure to music therapy may facilitate its understanding and implementation in clinical practice (Mercier et al. 2025; Carney et al. 2025). Consequently, by examining healthcare professionals' perceptions after direct experience with music therapy, this study provides a practice‐informed perspective on factors that may support its integration into routine hospital care.

Therefore, the purpose of this current study was to describe the perceptions of healthcare professionals regarding MT in terms of its: (1) Perceived benefits and clinical application of MT, and (2) Implementation and use of MT in hospital settings. In order to align the study objectives, the following specific research questions were formulated: What are the perceptions of healthcare professionals regarding the personal benefits and clinical application of active and receptive music therapy in hospital settings? How do healthcare professionals rate the implementation and continued use of music therapy in their workplace?

## 2 Methods

### 2.1 Design

This study was carried out using an exploratory descriptive design (Sandelowski 2000) as a naturalistic approach that has been used in other studies conducted in healthcare contexts. The qualitative description of a naturalistic approach studies and interprets the experiences of people in their natural state and social and cultural context (Armstrong 2010). The purpose is to produce a comprehensive and accurate descriptive summary of an everyday life event (Sandelowski 2000; Hunter et al. 2019).

In our study we sought to describe how healthcare professionals perceived MT and to identify the possible impact it can have on their needs, as well as on clinical practice. We provided a descriptive and comprehensive summary of the experiences healthcare professionals had in the interactive MT sessions and analysed, based on their experiences, whether its implementation was of benefit to quality of care. Therefore, the voice of healthcare professionals was privileged in our work because it was loaded with their self‐reflective constructions about the possible benefits of MT (Maldonado 2018).

### 2.2 Participants

The participants were selected for inclusion by convenience sampling (Elfil and Negida 2017). All healthcare professionals invited to participate accepted the invitation, resulting in a final sample of 22 participants. No participants withdrew from the study. The participant recruitment was carried out using the ‘snowballing’ technique whereby those who agreed to participate brought other professionals into the study. No prior relationship had been established between the researchers/facilitators and the participants before the study commenced. Thus, active medical and nursing professionals from different public and private hospitals in the province of Alicante (Spain) agreed to participate in this work. Gender parity was not considered because the recruitment was voluntary. As described in Table 1, information was collected on the main characteristics of the participants (their specialties, years worked in the healthcare field, age, sex, marital status, place of residence, hospital unit in which they worked, and knowledge of music, MT, or art therapy). The professionals came from different hospital units with high emotional exposure and high demand for care, including Paediatrics, Gynaecology, Internal Medicine, Psychiatry, Oncology and the Neonatal Intensive Care Unit. These services are characterised by continuous contact with patients in situations of clinical and/or emotional vulnerability, frequent decision‐making under pressure, and the need to sustain therapeutic links in contexts of organisational stress. This care setting constitutes an environment prone to emotional exhaustion and perceived overload, factors that may influence self‐care needs and receptiveness to non‐pharmacological interventions such as MT. The presence of different professional profiles (medical specialists, residents, nurses, and auxiliary technicians) made it possible to capture different perceptions depending on the type of clinical responsibility and the level of exposure to patient suffering.

**TABLE 1:** The sociodemographic characteristics and knowledge of music, music therapy, and art therapy of the healthcare professionals included in this work.

22 Healthcare professionals	
Specialties *n* (%)	
Doctors	8 (36.3%)
Medical intern residents	2 (9.0%)
Nurses	10 (45.4%)
Auxiliary care nursing technicians (ACNTs)	2 (9.0%)
Work unit	
Paediatric Department	9 (36.3%)
Gynaecology Department	4 (18.1%)
Internal Medicine Department	2 (9.0%)
Neonatal Intensive Care Unit	5 (22.7%)
Psychiatry Department	1 (4.5%)
Adult Oncology Section	1 (4.5%)
Years worked in healthcare fields	
≤ 10 years	2 (9.0%)
11–20 years	12 (54.5%)
21–30 years	6 (27.2%)
≥ 31 years	2 (9.0%)
Age	
25–35 years	6 (27.2%)
36–46 years	10 (45.4%)
47–57 years	5 (22.7%)
≥ 58 years	1 (4.5%)
Sex	Male *n* = 10 (45.4%) Female *n* = 12 (54.5%)
Marital status	Married *n* = 18 (81.8%) Divorced *n* = 2 (11.1%) Domestic partner *n* = 2 (11.1%)
Place of residence	Alicante *n* = 22 (100%)
Knowledge of music, music therapy, or art therapy	Music education *n* = 7 (38.8%)

All the healthcare professionals were explained the purpose of the study and the fact that they could withdraw from the research, with no negative consequences if they no longer wanted to participate in it. Finally, all the participants signed their informed consent.

### 2.3 Data Collection

Focus groups were chosen to explore the perceptions of healthcare professionals of MT. In this study, two focus groups were formed, each comprising 11 healthcare professionals. This technique generates questions that support the research objectives of a given project, allowing assessment of participants' responses and reviewing their similarities and differences (Halkier 2010). Furthermore, its application has proven useful for investigating the knowledge, norms, and values of certain groups, allowing access to a source of information rich in qualitative data (Hamui‐Sutton and Varela‐Ruiz 2013). Focus groups, rather than individual interviews, can produce deep and consistent knowledge about the phenomenon under study (Krueger and Casey 2015; Papastavrou and Andreou 2012). Conducting focus groups allowed us to create an opinion space to capture the thoughts, feelings, and actions of the participants, in addition to stimulating self‐explanation, in order to obtain qualitative data on the benefits of using MT (Hamui‐Sutton and Varela‐Ruiz 2013).

### 2.4 Procedure

The work with both focus groups was carried out in November 2023 in a classroom in the University of Alicante (Spain), Faculty of Health Sciences. Each of the group sessions lasted approximately 150 min and took place in three phases, each one lasting approximately 50 min. The sessions were moderated by the first author (R.C.R.), a male accredited music therapist. The second author (A.L.N.), a female researcher with extensive experience in qualitative research, acted as an assistant moderator (Halkier 2010). The role of the assistant moderator was to support the moderator by asking probing questions, observing the group interaction, taking field notes, and evaluating the moderator's interview technique (Krueger and Casey 2015). No other individuals were present during the focus group sessions.

As previously indicated, the focus groups were completed in three phases. In the first phase, a thematic guide containing ad hoc questions was used in order to understand the knowledge that healthcare professionals had about music, MT, or art therapy and if they had any specific sensitivities to music. For example, among others, they were asked the following questions: ‘Do any of you have any musical training, play a musical instrument, or have training in MT or art therapy?’ and ‘For you, what is the meaning of MT?’ In the second phase, MT techniques were implemented with healthcare professionals that involved both active participation (e.g., singing, playing musical instruments, and improvising) and receptive participation (listening to music) so they could directly experience the effects of MT for themselves. To implement the MT techniques, participants were invited to listen to a series of songs, some of them recorded and others performed live by the first author (R.C.R.). They were also offered percussion instruments so they could perform various improvisation exercises. The healthcare professionals sang several songs and the session was ended with a relaxation activity involving live music played on the piano by the first author (R.C.R.). More details of this are provided in Table 2.

**TABLE 2:** Music therapy session, second phase.

Techniques	Materials used
Listening to recorded songs and live renditions performed on the piano and saxophone by the first author	*My Light*—Román Rodríguez *Back in Black*—AC/DC *My Way*—Paul Anka *Quasi una fantasia*. Adagio—L. V. Beethoven *Flowers*—Miley Cyrus *Carmina Burana, O Fortuna*—Carl Orff *Bemba Colora*—Celia Cruz Digital piano Saxophone
Improvisation with percussion instruments (with question–answer and free improvisation exercises, coordinated by the first and second authors)	Tambourine, woodblock, maracas, güiro, cowbell, claves, rattles, drum, djembe, bongo, cabasa, and shaker
Singing (improvisation exercises with each student using their voice to sing their name and singing songs as a group, coordinated by the first and second authors)	*Imagine*—John Lennon *Vivir mi Vida*—Marc Anthony
Relaxation exercises with music (with prior adaptation through a breathing technique before listening to a live improvisation at a slow tempo in the keys of E− and G, performed by the first author on the piano)	Digital piano

The participants specifically evaluated each implemented technique and so were able to differentiate the effects of active MT (singing, improvisation, use of instruments) and receptive MT (listening to recorded or live music). This distinction allowed the professionals to identify the emotional, cognitive, and social effects of each intervention, thus improving their understanding of the context and applicability of MT in hospital settings.

It is worth noting that the session design was based on a multimodal approach to MT, integrating active and receptive techniques adapted to the needs of the participants, as described in Schapira et al. (2007). This approach allows each intervention to be individually tailored to therapeutic goals, combining improvisation, singing, instrument use, and listening to music to achieve simultaneous emotional, social, and cognitive effects.

To gather information about how the professionals had experienced the session, they were asked the following questions: Did listening to these songs affect you? Did playing percussion instruments have an effect on you? Did singing affect you? Did relaxing with music have an effect on you? What is your overall evaluation of the MT session? In the third phase, the healthcare professionals were first shown some video content about various MT interventions carried out in healthcare fields so they could learn about how MT interventions are carried out. Next, they were asked some questions about their opinions on the applicability of MT in clinical practice, as follows: Do you think MT can benefit patients, what types of benefits? Why? How do you think MT can change the care climate and hospital environment during the patient's stay? What is your assessment of the implementation of MT in hospital environments and why? All the questions asked in each of the phases are shown in Table 3. The information collected was audio recorded, and the interactions of each participant in the focus groups were transcribed verbatim.

**TABLE 3:** Focus group interview guide.

Introductory questions	Do any of you have any musical training, play an instrument, or have training in music therapy or art therapy, etc.? For you, what is the meaning of MT?
Frequent questions	How did listening to these songs affect you? What about when you were playing the percussion instruments? And when you were singing? How did relaxing with music affect you? What was your general evaluation of the MT session? Do you think MT can benefit patients? What patient types? Why? How do you think MT can change the care climate and hospital environment during patient stays? What is your opinion of the value of implementing MT in hospital environments and why?

Abbreviation: MT, music therapy.

### 2.5 Ethical Considerations

The study protocol was approved by the Ethics Committee of the University of Alicante (Spain) (reference: UA‐2019‐06‐12). The study was conducted in accordance with the ethical principles of the Declaration of Helsinki and its subsequent revisions. All participants provided written informed consent before participating in the study. In accordance with Spanish data protection legislation (Organic Law 3/2018 on the Protection of Personal Data and Guarantee of Digital Rights), all study records and signed informed consent forms were securely stored by the principal investigator, ensuring participant confidentiality. Participants were identified only by an assigned code.

### 2.6 Data Analysis

We used an inductive approach to analyse the data. Manifest qualitative content analysis was used to study the responses. This is best described as follows: “Manifest content analysis focuses on describing what is observable in a text, rather than interpreting meaning” (Bengtsson 2016).

We organised the data into significant themes according to concepts and themes that were recurring throughout, using the following four stages first identified by Bengtsson (2016): (1) decontextualisation: inductive coding identifying units of meaning in the data, (2) recontextualisation: comparing units of meaning with the textual data to ensure that all content has been covered in relation to the objectives and questions of the study, (3) categorisation: where homogeneous groups are condensed into categories or themes, and (4) compilation: where realistic conclusions and results can be drawn from the data (Bengtsson 2016).

Data generated from the transcription of the audio recordings of the focus groups were used to carry out the analysis and were read independently by two authors (R.C.R. and A.L.N.), who coded the data. We used MAXQDA software to prepare this analysis. To review the coherence, three researchers subsequently compared the similarities and differences of the codes identified, refining the themes and subthemes present in the participants' narratives. Any differences of opinion were discussed and the analysis was refined through consensus meetings between the investigators.

### 2.7 Research Rigour

In addition, we considered the four aspects of reliability (credibility, reliability, confirmability, and transferability) established by Guba and Lincoln (1994). The focus group sessions were fully transcribed and shared with the participants for their review and validation. This procedure ensured that their opinions and experiences were accurately represented and allowed for adjustments before the final analysis, thus increasing the credibility and rigour of the study. To improve the credibility of the findings, two researchers were present during the focus groups, which improved data collection. The credibility of the data in this study was maintained by continually comparing the data throughout the analysis process. It is important to consider that the research team comprised a music therapist, a paediatrician, and researchers with experience in qualitative studies, which allowed us to obtain a comprehensive overview during the data analysis (Nowell et al. 2017).

Sample adequacy was considered in relation to the aim of obtaining rich and diverse descriptions from healthcare professionals with different clinical roles and work settings. Data saturation was considered to have been achieved when no new codes or themes emerged during the analysis of the second focus group. At that point, the research team agreed that the data provided sufficient depth and variation to adequately address the research questions, consistent with current recommendations regarding saturation in qualitative research (Saunders et al. 2018).

Researcher reflexivity was actively considered throughout the study, particularly because the first author acted both as the accredited music therapist conducting the experiential sessions and as the moderator of the focus groups. The involvement of an experienced qualitative researcher as assistant moderator, together with the use of field notes, independent coding by two researchers, and consensus discussions within the research team, contributed to minimising the potential influence of the moderator's professional role on data collection and interpretation.

The validity and reliability of the work were achieved by carrying out a complete analysis of the data from the focus‐group information, which was contrasted with the field notes, thus obtaining a sufficiently dense description of the study phenomenon and achieving data saturation (Guba and Lincoln 1994; Noreña et al. 2012). Confirmability was maintained through the data coding and interpretation by creating a record of all the data. Additionally, the content of the focus groups and the extracted codes were given to two researchers familiar with qualitative research analysis methods and who had not participated in the study to verify the accuracy of the analysis process (Guba and Lincoln 1994). Transferability was ensured by providing as complete a description as possible of the topic, the characteristics of the participants, and examples of their statements and expressions (Ravenek and Rudman 2013). Additionally, we also followed the COREQ guidelines (Tong et al. 2007).

## 3 Results

A total of 22 healthcare professionals participated in this work. They were aged between 25 and 60 years and 12 were women and 10 were men (Table 1).

Based on our analysis, two main themes and five sub‐themes were identified. Table 4 describes how the themes, subthemes, and codes were organised.

**TABLE 4:** The perceptions of healthcare professionals regarding the music therapy session and its implementation.

Theme	Subtheme	Codes
Perceived benefits and clinical application of music therapy	Significant emotions and feelings	Happiness Sadness Nostalgia Hope Empowerment Group connection Calm Fun Disconnection Wellbeing Strength Companionship Evocation of memories Disconnection Relaxation
	Global perception of the music therapy session	Gratitude Reflection Satisfaction Introspection Sharing Worth
	Repercussions in the healthcare environment and professional wellbeing	Complement Nice atmosphere Dynamic atmosphere Relaxed atmosphere Job performance Interaction Benefits to workers
Implementation and use of music therapy in hospital settings	Music therapy as a therapeutic tool	Tool for a job Positive feedback
	Implementation of music therapy in hospital settings	Needs of patients Support from hospital management Qualified music therapist

### 3.1 Theme 1: Perceived Benefits and Clinical Application of Music Therapy

The first theme encompasses healthcare professionals' perceptions of the personal and emotional effects experienced after participating in the different music therapy techniques (music listening, singing, instrumental improvisation, and relaxation with music). Overall, participants' accounts reflected that the experience facilitated processes of introspection, emotional regulation, the evocation of personal memories, and interpersonal connection. Although the emotions expressed varied among participants, a common pattern emerged, characterised by increased emotional awareness and a positive evaluation of the experience. This theme was organised into two subthemes: (a) significant emotions and feelings and (b) global perception of the music therapy session, (c) repercussions in the healthcare environment and professional wellbeing.

#### 3.1.1 Significant Emotions and Feelings

Healthcare professionals described a range of emotional responses following the music listening activities. Although the emotions expressed varied among participants, their accounts consistently indicated that music facilitated introspection, the evocation of autobiographical memories, and the identification of both positive and negative emotional states. Overall, participants' accounts reflected increased emotional awareness and a deeper connection with personal and emotional experiences, highlighting the potential of music therapy to facilitate processes of emotional exploration. This experience was reflected in testimonies illustrating both the emotional changes elicited by music and the processes of personal reflection it facilitated:

The first song made me feel sadness and nostalgia, the second joy, and the third put me in a realistic and positive mood towards the future. (Participant 18)

Music made me stop and reflect on experiences that I rarely think about. It brought back personal memories and helped me recognise emotions that I usually set aside during my daily work. (Participant 5)

In addition, some participants indicated that the shared experience of listening to music and subsequently reflecting as a group fostered a more positive perception of their own personal resources. Overall, participants' accounts reflected increased self‐confidence and a greater perceived ability to cope with personal situations, showing how the experience contributed to strengthening their perception of their own emotional resources. This process was illustrated by the following testimony:

It made me realise that I am stronger than I thought. I left the session feeling that I could face challenges that had seemed overwhelming before. (Participant 1)

Active techniques, particularly improvisation with percussion instruments, were perceived as an opportunity to reduce mental tension, promote attention to the present moment, and strengthen interaction with colleagues. Participants emphasised the value of the shared process over the musical outcome, indicating that group synchrony and interaction during the activity fostered a sense of disconnection from the healthcare routine and connection with the group.

Playing gave me a sense of mental calm. I was able to connect, through rhythm, with my colleagues. (Participant 17)

Singing was described as an experience that facilitated emotional expression and contributed to improving mood. Participants indicated that singing allowed them to openly express their feelings and experience positive emotional changes. Some also highlighted that this activity helped them connect with emotions that usually remained in the background due to the demands of their professional practice.

Singing allowed me to express my feelings. My mood changed positively. (Participant 4)

In addition to facilitating emotional expression, singing evoked autobiographical memories and strengthened the sense of belonging to the group. Overall, participants described these experiences as moments of particular emotional significance, combining the evocation of personal experiences with connection with others. The following testimonies illustrate these findings.

When we sang the first song it transported me back to my childhood with my parents—a very pleasant feeling. (Participant 13)

Singing allowed me to perceive a feeling of camaraderie and, at times, I felt like crying with joy. (Participant 5)

Relaxation activities accompanied by music were perceived as an experience that promoted calm and reduced tension. Participants indicated that this intervention contributed to reducing stress and considered that it could be incorporated into hospital settings as a complement to conventional care.

I was able to relax and unwind and I think that music therapy is a very appropriate complement that can help patients a lot in terms of relaxation, among other things. (Participant 6)

#### 3.1.2 Global Perception of the Music Therapy Session

Healthcare professionals provided an overall positive evaluation of the music therapy session. Overall, they indicated that the experience allowed them to connect with themselves, reflect on personal and professional aspects, and become more aware of their own emotional state.

Thank you for this session, it allowed me to connect with my inner self. (Participant 3)

#### 3.1.3 Repercussions in the Healthcare Environment and Professional Wellbeing

Participants considered that music therapy could be a useful complement to clinical practice because of the benefits it may provide to patients. Overall, they highlighted its potential to promote emotional wellbeing and improve the hospitalisation experience, indicating that this type of intervention could be incorporated as part of routine care.

It could be a very interesting complement in clinical practice, offering moments of joy and relaxation, among many other benefits. (Participant 14)

Participants also considered that music therapy could contribute to creating a more relaxed hospital environment for patients, families, and healthcare professionals, fostering a more positive care environment. Overall, they indicated that this type of intervention could improve interpersonal relationships and wellbeing during hospitalisation, benefiting both those receiving care and those providing it.

Music therapy can help to dissipate tension and create a more relaxed working environment for patients, their families, and professionals. (Participant 6)

Participants considered that music therapy could strengthen interpersonal relationships and communication within the healthcare environment. Overall, they indicated that this type of intervention could foster a more positive working environment among healthcare professionals and improve the quality of interactions with patients, contributing to a more collaborative and humanised care environment.

…given that the work environment is essential to improving performance, few would doubt that music promotes positive feelings that stimulate camaraderie, work performance, and the wellbeing of patients and their families. (Participant 11)

The use of music therapy can create good interactions between patients and professionals and this, in turn, can benefit the healthcare delivery environment. (Participant 2)

Finally, healthcare professionals considered that music therapy could also benefit their own wellbeing by promoting a more positive mood and contributing to their personal and professional development. Overall, participants indicated that these benefits could have a positive impact on their clinical practice and on how they cope with the emotional demands of healthcare work.

Music therapy can change the state of mind of healthcare workers… favouring their personal and professional development. (Participant 9)

### 3.2 Theme 2: Implementation and Use of Music Therapy in Hospital Settings

This theme encompasses the perceptions of healthcare professionals regarding the practical application of music therapy in hospital settings. It includes two subthemes: (a) music therapy as a therapeutic tool and (b) implementation of music therap in hospital settings.

#### 3.2.1 Music Therapy as a Therapeutic Tool

The participants described MT as a therapeutic tool for work in the field of health service delivery because it allows the circumstances of patients, such as their emotional states, to be reviewed. This can sometimes be difficult for direct support healthcare staff, doctors, nurses, and other professionals involved in providing patient care.

In addition, participants considered music therapy to be a complementary intervention to conventional healthcare, as it may facilitate a broader understanding of patients' emotional needs. This perception was consistent across healthcare professionals, who highlighted its potential to promote more holistic and patient‐centred care.

I believe that music therapy can help us healthcare professionals learn more about the emotional states of patients, which is why I think it is a good tool. (Participant 7)

#### 3.2.2 Implementation of Music Therapy in Hospital Settings

All the healthcare professionals rated the implementation of MT in their hospital setting as positive, indicating that it would allow them to better understand some of the psychosocial needs of patients. For example, they said MT could help them collect information about how patients feel in relation to their illness. It could also directly influence how patients feel or cope with certain moments of stress during their hospital stay. Furthermore, it would also have repercussions on their quality of life by allowing them to channel their emotions.

Participants agreed that incorporating MT into hospital settings could contribute not only to improving patients' emotional wellbeing but also to promoting more humanised care focused on their psychosocial needs. They also indicated that its implementation could benefit healthcare professionals themselves by providing them with a better understanding of patients' experiences and needs during hospitalisation.

The implementation of music therapy would be a very powerful resource, both at a psychological level for the patients and their relatives, as well as to help healthcare personnel to understand the needs of each patient and be able to use this knowledge to improve patient stays, including their healing. (Participant 9)

Some professionals indicated that support from the hospital management would be required to implement this type of therapy. They acknowledged that this support is essential to mobilise resources, recognise the importance of music therapy, and disseminate the usefulness of these interventions.

In this regard, participants indicated that the implementation of music therapy depends not only on its acceptance by healthcare professionals but also on institutional commitment. They considered support from hospital management essential to promote organisational changes, allocate resources, and establish music therapy as part of routine clinical practice.

Changes in the organisation of hospital centres, especially in terms of mentality and awareness, not only of the management but also of the staff… Music therapy is necessary. (Participant 12)

In addition, several health professionals stated that it would be advisable for MT interventions to be performed by qualified professionals so that the techniques applied are appropriate for the intended purpose and are tailored to each individual patient.

Participants agreed that the effectiveness of music therapy depends on interventions being designed and implemented by professionals with specific training. They considered this qualification essential to adapt the techniques to the individual needs of each patient and ensure their appropriate application in hospital settings.

…but with a music therapist. There must be a good professional behind this technique to give each patient what they need. (Participant 15)

Although the main themes remained consistent among the participants, nuances were observed according to each professional's role and work context. For example, nurses and auxiliary technicians most frequently highlighted the immediate emotional support and sense of wellbeing generated during the sessions, while physicians valued their potential to improve patient care and communication. Likewise, professionals in units with a high emotional load such as Intensive Care or Oncology, tended to report more intense emotional responses.

Although these differences do not constitute generalisable patterns due to the qualitative nature of the study, they suggest that professional experience and the clinical context may shape perceptions of music therapy and the benefits attributed to it. Overall, these nuances enrich the understanding of the phenomenon under study by showing how the priorities and needs of each professional group influence their evaluation of this intervention.

## 4 Discussion

The objective of this study was to describe the perceptions of healthcare professionals regarding (1) the perceived benefits and clinical application of MT and (2) the implementation and use of MT in hospital settings. This section presents and discusses the findings according to these two main themes and their respective sub‐themes.

### 4.1 Perceived Benefits and Clinical Application of Music Therapy

This study explored healthcare professionals' perceptions after directly participating in experiential music therapy sessions, allowing their views to be analysed based on personal experience rather than solely on prior knowledge or expectations regarding this intervention. Overall, the findings show that the experience fostered a broader understanding of the potential of music therapy, not only as an intervention for patients but also as a resource capable of promoting reflection, emotional wellbeing, and a more favourable attitude towards its integration into hospital settings. These findings provide a complementary perspective to the existing literature by highlighting the importance of direct experience in shaping perceptions of the clinical usefulness of MT.

In our study, through the application of receptive MT techniques such as listening to songs, we aimed to get health professionals to identify and express different emotions and feelings evoked by the music. They expressed joy and sadness and feelings associated with nostalgia and hope, among others.

These findings suggest that receptive music therapy techniques facilitate the identification and expression of emotions, as well as processes of introspection and the evocation of personal experiences. One possible explanation for these effects is that music activates brain networks involved in emotional processing and affective regulation, facilitating the emergence of complex emotional and cognitive responses (Mas‐Herrero et al. 2018). It is particularly relevant that these responses were described by healthcare professionals, as direct experience may contribute to a deeper understanding of the emotional processes subsequently experienced by patients and foster a more receptive attitude towards the integration of music therapy into clinical practice.

These findings are consistent with previous studies showing that music therapy promotes psychosocial wellbeing, emotional expression, and creativity, while also contributing to symptom management in children and adults and improving their quality of life (Bradt et al. 2013; Drake et al. 2017; Rodríguez‐Rodríguez et al. 2023). Overall, this evidence supports the idea that music therapy can facilitate emotional regulation processes across different clinical settings and populations.

Participants described how active music therapy techniques, such as singing and improvisation with percussion instruments, facilitated the evocation of memories, connection with colleagues, and disconnection from the healthcare routine. Overall, these findings suggest that such activities not only promote individual wellbeing but also strengthen group interaction and create opportunities for emotional regulation within the healthcare environment. This interpretation is consistent with Odell‐Miller (2019), who noted that different music therapy techniques can be used for activation or relaxation purposes, promoting social, emotional, psychological, and cognitive benefits according to the needs of each context.

Consistent with these findings, participants positively valued receptive techniques aimed at relaxation, reporting that they provided a sense of calm and wellbeing. The positive evaluation of these techniques suggests that music therapy could be a useful tool for reducing the emotional tension associated with clinical practice, particularly in hospital settings characterised by a high emotional burden. These findings are consistent with those of Zamanifar et al. (2020), who observed a significant reduction in anxiety and work‐related stress among nursing professionals following an intervention based on music therapy and aromatherapy. Overall, this evidence suggests that music therapy may contribute to the emotional wellbeing of healthcare professionals, in addition to the benefits previously described for patients.

### 4.2 Implementation and Use of Music Therapy in Hospital Settings

Healthcare professionals considered that music therapy could be a useful tool in clinical practice, not only because of its potential benefits for patients but also because of its capacity to promote a more positive and dynamic working environment. These findings suggest that music therapy could contribute to humanising the hospital environment by promoting closer relationships between healthcare professionals and patients and fostering a healthier care environment.

This interpretation is consistent with the findings reported by Patterson et al. (2015), who observed that healthcare professionals positively valued patients' participation in music therapy programmes and reported greater job satisfaction when sharing musical activities with them. Similarly, different studies have indicated that music therapy may contribute to reducing work‐related stress and improving mood, attention, concentration, and professional performance (Raglio et al. 2017; Zamanifar et al. 2020). In settings with a high care burden, it has also been reported to reduce fatigue, sadness, fear, and worry among healthcare professionals (Giordano et al. 2020).

Overall, our findings extend this evidence by showing that healthcare professionals perceive music therapy not only as an intervention for patients but also as a resource that may improve the working environment and promote the emotional wellbeing of healthcare teams.

## 5 Limitations

This study was only carried out with health professionals from a single province in Spain. In future work, it would be interesting to record and compare the perceptions of MT of health professionals from different geographical locations. Furthermore, although the data collected from the focus group sample was sufficient and allowed for data saturation, it would be valuable to conduct future research with a mixed method approach and to combine both qualitative and quantitative methodologies. Furthermore, our sample mainly comprised women and so it is possible that different results would be found in a sample more homogeneous by sex. The fact that the ages of the participants were distributed over a wide range could also have influenced the results. Thus, further research will be needed to shed light on the full value of MT.

Finally, the experiential nature of the intervention should be considered when interpreting the findings. Participants shared their perceptions immediately after taking part in an active and receptive music therapy session, and this direct experience may have positively influenced their views of music therapy. Although this experiential approach represents one of the strengths of the study by providing first‐hand experience, it may also constitute a potential source of bias when interpreting participants' perceptions.

## 6 Conclusions and Implications for Practice

Participation in experiential music therapy sessions allowed healthcare professionals to directly experience different active and receptive techniques, facilitating processes of introspection, emotional expression, the evocation of memories, and reflection on personal and professional aspects. Based on this experience, participants perceived music therapy as an intervention with the potential to promote emotional wellbeing, improve communication, and contribute to the humanisation of care in hospital settings. They also considered that its integration as a complement to conventional care could be valuable, provided that the interventions are delivered by qualified music therapists.

As these findings are based on healthcare professionals' perceptions and experiences, they should be interpreted with caution and not as evidence of demonstrated clinical effects on patients or the quality of care. Nevertheless, this study provides relevant qualitative evidence on how direct experience with music therapy may contribute to a broader understanding of this intervention among healthcare professionals and foster a more receptive attitude towards its potential integration into hospital settings. These findings provide a useful basis for future research evaluating the impact of music therapy through designs that also allow its clinical and organisational effects to be examined.

## Author Contributions

**Román‐Carlos Rodríguez Rodríguez:** methodology, investigation, formal analysis, writing – original draft, writing – review and editing. **Ana Noreña Peña:** methodology, formal analysis, supervision, writing – original draft, writing – review and editing, investigation. **Teresa Chafer Bixquert:** writing – review and editing, writing – original draft. **Javier González de Dios:** methodology, formal analysis, data curation, supervision. **Carmen Solano Ruiz:** conceptualization, formal analysis, writing – review and editing, writing – original draft.

## Funding

The authors have nothing to report.

## Conflicts of Interest

The authors declare no conflicts of interest.

## Supporting information


**Data S1:** COREQ (COnsolidated criteria for REporting Qualitative research) Checklist

## Data Availability

The data that support the findings of this study are available from the corresponding author upon reasonable request. The data are not publicly available due to privacy and ethical restrictions.

## References

[nop270934-bib-0001] Alzahrani, N. 2021. “The Effect of Hospitalization on Patients' Emotional and Psychological Well‐Being Among Adult Patients: An Integrative Review.” Applied Nursing Research: ANR 61: 151488. 10.1016/j.apnr.2021.151488. 34544571

[nop270934-bib-0002] Armstrong, J. 2010. “Naturalistic Inquiry.” In Encyclopedia of Research Design, edited by N. J. Salk . Sage.

[nop270934-bib-0003] Bengtsson, M. 2016. “How to Plan and Perform a Qualitative Study Using Content Analysis.” NursingPlus Open 2: 8–14. 10.1016/j.npls.2016.01.001.

[nop270934-bib-0004] Biard, M. , M. Brinkley , H. Gómez , M. S. Collinson Niestrawski , and J. Hagan . 2026. “Evaluation of Staff Perceptions of Music Therapy in a Medical Setting.” Music Therapy Perspectives 43, no. 2: miaf015. 10.1093/mtp/miaf015.

[nop270934-bib-0005] Borick, K. M. , J.‐M. Stacciarini , J. J. Tanner , et al. 2026. “Music in Care (MIC) by Nurses: Initial Validation of a Questionnaire to Characterize Nurses' Attitudes and Feasibility of Implementation on the Use of Music in Patient Care.” Journal of Holistic Nursing 44, no. 1: 7–21. 10.1177/08980101241276907. PMC11896893 39263724

[nop270934-bib-0006] Bradt, J. , C. Dileo , K. Myers‐Coffman , and J. Biondo . 2021. “Music Interventions for Improving Psychological and Physical Outcomes in People With Cancer.” Cochrane Database of Systematic Reviews 2021, no. 10: CD006911. 10.1002/14651858.CD006911.pub4. PMC8510511 34637527

[nop270934-bib-0007] Bradt, J. , C. Dileo , and M. Shim . 2013. “Music Interventions for Preoperative Anxiety.” Cochrane Database of Systematic Reviews 2013, no. 6: CD006908. 10.1002/14651858.CD006908.pub2. PMC9758540 23740695

[nop270934-bib-0008] Carney, K. A. , R. M. Wiste , S. M. Cutshall , et al. 2025. “Palliative Care Staff Attitudes Toward Music Therapy for Hospitalized Adult Patients.” American Journal of Hospice & Palliative Medicine: 10499091251407097. 10.1177/10499091251407097. 41408945

[nop270934-bib-0009] Carter, A. M. , E. R. Dioso , B. Romero , C. E. Clinker , and B. Lucke‐Wold . 2023. “Complementary Medicine and Expressive Arts Therapy: Adjuvant for Recovery Following Neurosurgical Procedures.” OBM Integrative and Complementary Medicine 8, no. 1: 1–14. 10.21926/obm.icm.2301007.

[nop270934-bib-0010] Chadder, N. E. M. 2019. “An Exploration Into the Perception of Music Interventions in Hospitals Amongst Healthcare Professionals.” Voices: A World Forum for Music Therapy 19, no. 1. 10.15845/voices.v19i1.2711.

[nop270934-bib-0011] Cibrian, F. L. , M. Madrigal , M. Avelais , and M. Tentori . 2020. “Supporting Coordination of Children With ASD Using Neurological Music Therapy: A Pilot Randomized Control Trial Comparing an Elastic Touch‐Display With Tambourines.” Research in Developmental Disabilities 106: 103741. 10.1016/j.ridd.2020.103741. 32950853

[nop270934-bib-0012] Drake, L. , F. Ben‐Yelun , and M. J. del Olmo . 2017. “Musicoterapia en Una UCI pediátrica: El Uso de la Música Como Emergente Emocional en el Proceso de intervención Con Niños en Situación de Riesgo [Music Therapy in a Paediatric ICU: The Use of Music as an Emotional Emergent in the Intervention Process With Children at Risk].” Revista de Investigación en Musicoterapia 1: 14–31. 10.15366/rim2017.1.002.

[nop270934-bib-0013] Elfil, M. , and A. Negida . 2017. “Sampling Methods in Clinical Research: An Educational Review.” Emergency (Tehran) 5, no. 1: e52. PMC5325924 28286859

[nop270934-bib-0014] European Music Therapy Confederation . n.d. “Recognition.” Accessed August 19, 2026. https://emtc‐eu.com/recognition/.

[nop270934-bib-0015] Facchini, M. , and C. Ruini . 2021. “The Role of Music Therapy in the Treatment of Children With Cancer: A Systematic Review of Literature.” Complementary Therapies in Clinical Practice 42: 101289. 10.1016/j.ctcp.2020.101289. 33316592

[nop270934-bib-0016] Ford, K. , L. Tesch , J. Dawborn , and H. Courtney‐Pratt . 2018. “Art, Music, Story: The Evaluation of a Person‐Centred Arts in Health Programme in an Acute Care Older Persons' Unit.” International Journal of Older People Nursing 13, no. 2: e12186. 10.1111/opn.12186. 29363263

[nop270934-bib-0017] Giordano, F. , E. Scarlata , M. Baroni , et al. 2020. “Receptive Music Therapy to Reduce Stress and Improve Wellbeing in Italian Clinical Staff Involved in COVID‐19 Pandemic: A Preliminary Study.” Arts in Psychotherapy 70: 101688. 10.1016/j.aip.2020.101688. PMC7361107 32834302

[nop270934-bib-0018] Giordano, F. , T. Trevor‐Briscoe , C. Rutigliano , et al. 2025. “A Multicentric Survey of the Experience and Perception of Medical Music Therapy Among Health Professionals in Italian AIEOP Paediatric Cancer Centres.” Arts in Psychotherapy 95: 102331. 10.1016/j.aip.2025.102331.

[nop270934-bib-0019] Guba, E. G. , and Y. S. Lincoln . 1994. “Competing Paradigms in Qualitative Research.” In Handbook of Qualitative Research, edited by N. K. Denzin and Y. S. Lincoln , 105–117. Sage.

[nop270934-bib-0020] Halkier, B. 2010. “Focus Groups as Social Enactments: Integrating Interaction and Content in the Analysis of Focus Group Data.” Qualitative Research 10, no. 1: 71–89. 10.1177/1468794109348683.

[nop270934-bib-0021] Hamui‐Sutton, A. , and M. Varela‐Ruiz . 2013. “The Focal Groups' Technique.” Investigación en Educación Médica 2, no. 5: 55–60. 10.1016/S2007-5057(13)72683-8.

[nop270934-bib-0022] Hu, J. , J. Zhang , L. Hu , H. Yu , and J. Xu . 2021. “Art Therapy: A Complementary Treatment for Mental Disorders.” Frontiers in Psychology 12.: 686005. 10.3389/fpsyg.2021.686005. PMC8397377 34456801

[nop270934-bib-0023] Hunter, D. J. , J. McCallum , and D. Howes . 2019. “Defining Exploratory‐Descriptive Qualitative (EDQ) Research and Considering Its Application to Healthcare.” Journal of Nursing and Health Care 4, no. 1. http://eprints.gla.ac.uk/180272/.

[nop270934-bib-0024] Kennedy, H. , K. Reed , and M. Z. Wamboldt . 2014. “Staff Perceptions of Complementary and Alternative Therapy Integration Into a Child and Adolescent Psychiatry Program.” Arts in Psychotherapy 41, no. 1: 21–26. 10.1016/j.aip.2013.10.007.

[nop270934-bib-0025] Kern, P. , and D. B. Tague . 2017. “Music Therapy Practice Status and Trends Worldwide: An International Survey Study.” Journal of Music Therapy 54, no. 3: 255–286. 10.1093/jmt/thx011. 28992203

[nop270934-bib-0026] Krueger, R. A. , and M. A. Casey . 2015. Focus Groups: A Practical Guide for Applied Research. 5th ed. Sage.

[nop270934-bib-0027] Lane, D. , J. B. Palmer , and Y. Chen . 2019. “A Survey of Surgeon, Nurse, Patient, and Family Perceptions of Music and Music Therapy in Surgical Contexts.” Music Therapy Perspectives 37, no. 1: 28–36. 10.1093/mtp/miy008.

[nop270934-bib-0028] Li, X. , L. He , J. Wang , and M. Wang . 2019. “Illness Uncertainty, Social Support, and Coping Mode in Hospitalized Patients With Systemic Lupus Erythematosus in a Hospital in Shaanxi, China.” PLoS One 14, no. 2: e0211313. 10.1371/journal.pone.0211313. PMC6383916 30789919

[nop270934-bib-0029] Liu, Q. , W. Li , Y. Yin , et al. 2022. “The Effect of Music Therapy on Language Recovery in Patients With Aphasia After Stroke: A Systematic Review and Meta‐Analysis.” Neurological Sciences 43, no. 2: 863–872. 10.1007/s10072-021-05743-9. 34816318

[nop270934-bib-0030] Livingston, G. , L. Kelly , E. Lewis‐Holmes , et al. 2014. “A Systematic Review of the Effectiveness and Cost‐Effectiveness of Sensory, Psychological and Behavioural Interventions for Managing Agitation in Older Adults With Dementia.” Health Technology Assessment 18, no. 39: 1–226. 10.3310/HTA18390. PMC4781145 24947468

[nop270934-bib-0031] Maldonado, E. 2018. Metodología de la Investigación Social: Paradigmas: Cuantitativo, Sociocrítico, Cualitativo, Complementario [Social Research Methodology: Paradigms: Quantitative, Socio‐Critical, Qualitative, Complementary]. Ediciones de la U.

[nop270934-bib-0032] March‐Luján, V. A. 2022. “Musicoterapia en el S. XXI en España: Situación y Evolución Profesional, Formativa y Asociativa [Music Therapy in the 21st Century in Spain: Professional, Training, and Associative Situation and Development].” Brazilian Journal of Music Therapy 32: 74–99. 10.51914/brjmt.32.2021.386.

[nop270934-bib-0033] Mas‐Herrero, E. , A. Dagher , and R. J. Zatorre . 2018. “Modulating Musical Reward Sensitivity Up and Down With Transcranial Magnetic Stimulation.” Nature Human Behaviour 2, no. 1: 27–32. 10.1038/s41562-017-0241-z. 30980048

[nop270934-bib-0034] Meghani, N. , M. F. Tracy , S. O'Conner‐Von , N. N. Hadidi , M. A. Mathiason , and R. Lindquist . 2020. “Generating Evidence of Critical Care Nurses' Perceptions, Knowledge, Beliefs, and Use of Music Therapy, Aromatherapy, and Guided Imagery.” Dimensions of Critical Care Nursing 39, no. 1: 47–57. 10.1097/DCC.0000000000000391. 31789985

[nop270934-bib-0035] Mercier, L. J. , D. M. Langelier , J. Buchanan , S. Robinson , and S. Plamondon . 2025. “Development and Integration of a Music Therapy Program in the Neurologic Inpatient Setting: A Qualitative Study.” Disability and Rehabilitation 47, no. 9: 2304–2313. 10.1080/09638288.2024.2393439. 39183697

[nop270934-bib-0036] Moreno‐Morales, C. , R. Calero , P. Moreno‐Morales , and C. Pintado . 2020. “Music Therapy in the Treatment of Dementia: A Systematic Review and Meta‐Analysis.” Frontiers in Medicine 7: 1–11. 10.3389/fmed.2020.00160. PMC7248378 32509790

[nop270934-bib-0037] Noreña, A. , N. Alcaraz‐Moreno , J. G. Rojas , and R. Rebolledo Malpica . 2012. “Applicability of the Criteria of Rigor and Ethics in Qualitative Research.” Aquichan 12, no. 3: 263–274. 10.5294/aqui.2012.12.3.5.

[nop270934-bib-0038] Nowell, L. S. , J. M. Norris , D. E. White , and N. J. Moules . 2017. “Thematic Analysis: Striving to Meet the Trustworthiness Criteria.” International Journal of Qualitative Methods 16, no. 1: 1–13. 10.1177/1609406917733847.

[nop270934-bib-0039] Odell‐Miller, H. 2019. A Comprehensive Guide to Music Therapy: Theory, Clinical Practice, Research and Training. Jessica Kingsley Publishers.

[nop270934-bib-0040] Papastavrou, E. , and P. Andreou . 2012. “Exploring Sensitive Nursing Issues Through Focus Group Approaches.” Health Science Journal 6, no. 2: 185–200.

[nop270934-bib-0041] Patterson, S. , M. Duhig , C. Darbyshire , R. Counsel , N. Higgins , and I. Williams . 2015. “Implementing Music Therapy on an Adolescent Inpatient Unit: A Mixed‐Methods Evaluation of Acceptability, Experience of Participation and Perceived Impact.” Australasian Psychiatry 23, no. 5: 556–560. 10.1177/1039856215592320. 26129812

[nop270934-bib-0042] Raglio, A. , C. Imbriani , and E. Oddone . 2017. “Musicoterapia e Medicina del Lavoro [Music Therapy and Occupational Medicine].” Giornale Italiano di Medicina del Lavoro ed Ergonomia 38, no. 4: 257–260. 29916606

[nop270934-bib-0043] Ravenek, M. J. , and D. L. Rudman . 2013. “Bridging Conceptions of Quality in Moments of Qualitative Research.” International Journal of Qualitative Methods 12, no. 1: 436–456. 10.1177/160940691301200122.

[nop270934-bib-0044] Rodríguez‐Rodríguez, R.‐C. , A. Noreña‐Peña , T. Chafer‐Bixquert , J. González de Dios , A. I. Gutiérrez García , and C. Solano Ruiz . 2023. “The Value of Music Therapy in the Expression of Emotions in Children With Cancer.” European Journal of Cancer Care 2023: 2910350. 10.1155/2023/2910350.

[nop270934-bib-0045] Rodríguez‐Rodríguez, R.‐C. , A. Noreña‐Peña , T. Chafer‐Bixquert , A. Lorenzo Vásquez , J. González de Dios , and C. Solano Ruiz . 2022. “The Relevance of Music Therapy in Paediatric and Adolescent Cancer Patients: A Scoping Review.” Global Health Action 15, no. 1: 1–12. 10.1080/16549716.2022.2116774. PMC9543118 36174131

[nop270934-bib-0046] Sandelowski, M. 2000. “Focus on Research Methods: Whatever Happened to Qualitative Description?” Research in Nursing & Health 23, no. 4: 334–340. 10.1002/1098-240X(200008)23:4<334::AID-NUR9>3.0.CO;2-G. 10940958

[nop270934-bib-0047] Saunders, B. , J. Sim , T. Kingstone , et al. 2018. “Saturation in Qualitative Research: Exploring Its Conceptualization and Operationalization.” Quality & Quantity 52, no. 4: 1893–1907. 10.1007/s11135-017-0574-8. PMC5993836 29937585

[nop270934-bib-0048] Schapira, D. , K. Ferrari , V. Sánchez , and M. Hugo . 2007. Musicoterapia: Abordaje Plurimodal. ADIM Ediciones.

[nop270934-bib-0049] Silverman, M. J. , and J. Bibb . 2018. “Acute Care Mental Health Workers' Assumptions and Expectations of Music Therapy: A Qualitative Investigation.” Arts in Psychotherapy 59: 94–100. 10.1016/j.aip.2018.05.002.

[nop270934-bib-0050] Stegemann, T. , M. Geretsegger , E. Phan Quoc , H. Riedl , and M. Smetana . 2019. “Music Therapy and Other Music‐Based Interventions in Pediatric Health Care: An Overview.” Medicine 6, no. 1: 25. 10.3390/medicines6010025. PMC6473587 30769834

[nop270934-bib-0051] Tong, A. , P. Sainsbury , and J. Craig . 2007. “Consolidated Criteria for Reporting Qualitative Research (COREQ): A 32‐Item Checklist for Interviews and Focus Groups.” International Journal for Quality in Health Care 19, no. 6: 349–357. 10.1093/intqhc/mzm042. 17872937

[nop270934-bib-0052] Trainor, H. 2019. “Effects of Using Music Therapy for Patients Suffering From Dementia.” Health Care Manager 38, no. 3: 206–210. 10.1097/HCM.0000000000000276. 31261197

[nop270934-bib-0053] Tucquet, B. , and M. Leung . 2014. “Music Therapy Services in Pediatric Oncology.” Journal of Pediatric Oncology Nursing 31, no. 6: 327–338. 10.1177/1043454214533424. 25027188

[nop270934-bib-0054] Uggla, L. , K. Mårtenson Blom , L. O. Bonde , B. Gustafsson , and B. Wrangsjö . 2019. “An Explorative Study of Qualities in Interactive Processes With Children and Their Parents in Music Therapy During and After Pediatric Hematopoietic Stem Cell Transplantation.” Medicine 6, no. 1: 28. 10.3390/medicines6010028. PMC6473402 30781617

[nop270934-bib-0055] Umbrello, M. , T. Sorrenti , G. Mistraletti , P. Formenti , D. Chiumello , and S. Terzoni . 2019. “Music Therapy Reduces Stress and Anxiety in Critically Ill Patients: A Systematic Review of Randomized Clinical Trials.” Minerva Anestesiologica 85, no. 8: 886–898. 10.23736/S0375-9393.19.13526-2. 30947484

[nop270934-bib-0056] Wilson, C. , H. Bungay , C. Munn‐Giddings , and M. Boyce . 2015. “Healthcare Professionals' Perceptions of the Value and Impact of the Arts in Healthcare Settings: A Critical Review of the Literature.” International Journal of Nursing Studies 56: 90–101. 10.1016/j.ijnurstu.2015.11.003. 26696399

[nop270934-bib-0057] World Federation of Music Therapy . 2026. “A New Definition of Music Therapy.” https://www.wfmt.info/post/a‐new‐definition‐of‐music‐therapy‐july‐2026.

[nop270934-bib-0058] Zamanifar, S. , M. I. Bagheri‐Saveh , A. Nezakati , R. Mohammadi , and J. Seidi . 2020. “The Effect of Music Therapy and Aromatherapy With Chamomile‐Lavender Essential Oil on the Anxiety of Clinical Nurses: A Randomized and Double‐Blind Clinical Trial.” Journal of Medicine and Life 13, no. 1: 87–93. 10.25122/jml-2019-0105. PMC7175441 32341707

